# Pioglitazone Effect on Glioma Stem Cell Lines: Really a Promising Drug Therapy for Glioblastoma?

**DOI:** 10.1155/2016/7175067

**Published:** 2016-05-25

**Authors:** Chiara Cilibrasi, Valentina Butta, Gabriele Riva, Angela Bentivegna

**Affiliations:** ^1^School of Medicine and Surgery, University of Milano-Bicocca, Via Cadore 48, 20900 Monza, Italy; ^2^Ph.D. Program in Neuroscience, University of Milano-Bicocca, Via Cadore 48, 20900 Monza, Italy; ^3^NeuroMI, Milan Center of Neuroscience, Department of Neurology and Neuroscience, San Gerardo Hospital, Via Pergolesi 33, 20052 Monza, Italy

## Abstract

Glioblastoma multiforme (GBM) represents one of the most frequent malignant brain tumors. Current therapies do not provide real solutions to this pathology. Their failure can be ascribed to a cell subpopulation with stem-like properties called glioma stem cells (GSCs). Therefore, new therapeutic strategies GSC-targeted are needed. PPAR*γ*, a nuclear receptor involved in lipid metabolism, has already been indicated as a promising target for antineoplastic therapies. Recent studies have reported that synthetic PPAR*γ* agonists, already in clinical use for the treatment of type II diabetes, exhibit antineoplastic effects in a wide range of malignant tumor cells, including glioma cells. We investigated the effect of the synthetic PPAR*γ* agonist Pioglitazone on viability, proliferation, morphology, and differentiation in six GSC lines isolated from GBM patients. We also analyzed Pioglitazone-induced changes in transcriptional levels of Wnt/*β* catenin related genes. Results showed that response to Pioglitazone was heterogeneous inducing an evident decrease of cell viability and proliferation only in a subset of GSC lines. We did not find any sign of cell differentiation neither observing cell morphology nor analyzing the expression of stemness and differentiation markers. Moreover, Wnt/*β* signaling pathway was only mildly affected from a transcriptional point of view after Pioglitazone exposure.

## 1. Introduction

Glioblastoma multiforme (GBM; WHO grade IV) is the most common and aggressive form of brain tumors in adults. Despite improvements in treatment outcome the survival rates are still very poor, with only one-third of patients alive after one year [1].

Increasing evidence suggests that glioma stem cells (GSCs) are likely to play a key role in GBM onset and account for resistance to standard therapies and tumor recurrence [2, 3]. To date there is still no treatment available that can successfully eradicate the GSC subpopulation; indeed the research of new GSC therapeutic targets is needed in order to really improve GBM patients survival.

PPAR*γ* is a ligand-activated transcription factor belonging to the steroid/thyroid nuclear receptors family. In particular, it is involved in lipid metabolism [4] and its expression is induced during adipogenesis and required for fat cells terminal differentiation [5]. Upon activation due to interaction with cognate ligands, such as long chain polyunsaturated fatty acids [6] or prostaglandin [7], PPAR*γ* moves to the nucleus where it forms a heterodimer with retinoid X receptors (RXR). Then, this complex binds to peroxisome proliferator response elements (PPRE), leading to transcriptional activation of target genes [8].

A wide range of synthetic PPAR*γ* ligands have been identified, and some of these, such as the thiazolidinediones (Pioglitazone, Rosiglitazone), are in clinical use as antidiabetes drugs [9].

Intriguingly, PPAR*γ* agonists have also been found to be of interest in cancer treatment [10]. In particular, the activation of PPAR*γ*, which is expressed in high grade gliomas, has been shown to have several antineoplastic effects on human and rat glioma cell lines, inducing growth arrest and apoptosis* in vitro* and* in vivo* [11, 12] and inhibiting CD133+ cells expansion [13]. Moreover, a retrospective study has demonstrated that diabetic GBM patients treated with thiazolidinedione drugs had an increased median survival [14], suggesting that PPAR*γ* could represent a novel potential therapeutic target for the treatment of high grade glioma.

In this study we analyzed* in vitro* the effects of Pioglitazone exposure on cell viability and proliferation in six GSC lines isolated from GBM. We investigated its effect on stemness and differentiation through the expression of specific markers. Finally, since Wnt/*β* catenin pathway is aberrantly activated in cancer stem cells [15, 16] and Pioglitazone inhibits *β* catenin expression in glioma cells [17], for the first time to our knowledge we extended the analysis of this pathway analyzing the expression levels of seven related genes.

## 2. Materials and Methods

### 2.1. Cell Lines and Cell Culture Conditions

All the GSC lines used in this work (GBM2, G144, G179, G166, GliNS2, and GBM04) were isolated from patients affected by GBM and extensively characterized for their stem cell properties. GBM2, GBM7, G144, G166, GliNS2, and GBM04 derived from classic glioblastoma multiforme, while G179 derived from a giant cell variant glioblastoma. All the GSC lines have been already expanded* in vitro* as stable cell lines and used as powerful model for studying their biology and testing drug susceptibility [18, 19]. In 2013, our research group characterized their cytogenomic and epigenomic profiles [20]. The stemness properties of the GSC lines were periodically monitored, as already described in Baronchelli et al. 2013 [20]. Cell expansion was carried out in a proliferation permissive medium composed by DMEM F-12 (Euroclone) and Neurobasal 1 : 1 (Invitrogen), B-27 supplement without vitamin A (Invitrogen), 2 mM L-glutamine (Euroclone), 10 ng/mL recombinant human bFGF and 20 ng/mL recombinant human EGF (Miltenyi Biotec), and 20 UI/mL penicillin and 20 *μ*g/mL streptomycin (Euroclone) (complete medium). GSCs were cultured in adherent culture condition in T-25 cm^2^ flasks coated with 10 *μ*g/mL laminin (Invitrogen), in 5% CO_2_/95% O_2_ atmosphere.

### 2.2. Drugs and Treatments

Pioglitazone (Pio) (Cayman Chemical) was dissolved in dimethyl sulfoxide (DMSO) to make a 20 mM stock solution and then diluted to the final selected concentration (1-10-30-50 *μ*M) with PBS 1x. The stock preparation was stored at −20°C. DMSO had no effect on the cell survival.

### 2.3. MTT Assay

Cell metabolic activity was assessed by the MTT (3-[4,5-dimethylthiazol-2-yl]-2,5-diphenyl tetrazolium bromide) assay in order to evaluate the efficacy of Pio (1-10-30-50 *μ*M) treatment. Cells were plated in 96-well plates at a density of 4 × 10^4^ cells/well in 100 *μ*L of culture medium and incubated at 37°C. After 24 hs, Pio was added to cell culture medium. After the drug incubation time (24, 48, or 72 hs) MTT solution (1 mg/mL, Sigma) was added to each well and cells were incubated for 3 hs at 37°C. Therefore, formazan was solubilized in absolute ethanol and the absorbance of the dye was measured spectrophotometrically with the FLUOstar Omega microplate reader (BMG Labtech) at 595 nm. The percentage of inhibition was determined by comparing the absorbance values of drug-treated cells with that of untreated controls: [(treated-cell absorbance/untreated cell absorbance) × 100]. The results reported are the mean values of two different experiments performed at least in triplicate.

### 2.4. Cytomorphological Analysis

Cells were plated in T-25 cm^2^. When cells reached the 80% confluence, they were treated with Pio 10 *μ*M 48 hs. Subsequently, chromosomal preparations were performed by means of standard procedures as previously described [20]. Briefly, chromosomes were QFQ-banded using quinacrine mustard and slides were mounted in McIlvaine buffer. Slides were analyzed using Nikon Eclipse 80i fluorescence microscope (Nikon) (60x magnification) equipped with a COHU High Performance CCD camera. Mitotic index (MI) was evaluated counting the percentage of mitosis scoring at least 1000 nuclei. Data were obtained as mean values, derived from two independent experiments using cells at different passages.

For cell morphology studies, cells were plated in 6-well plates without laminin coating in proliferative permissive medium at 3 × 10^3^–10^4^ cells/mL, depending on the growth rate of each cell line, and after 24 hs, they were treated with Pio (1-10-30-50 *μ*M) for different times (24, 48, and 72 hs). The presence of morphological changes was estimated through the observation at phase contrast microscopy, comparing Pio-treated and untreated cells. Representative images were taken for each cell line and for each treatment.

### 2.5. Immunofluorescence

The immunofluorescence assay was performed on untreated and Pio 10 *μ*M treated cultures for 72 hs. The experiments were performed on all the GSC lines using mouse anti-PPAR*γ* (Santa Cruz Biotechnology, Santa Cruz, CA, USA; 1 : 50), rabbit anti-CD133 (Santa Cruz Biotechnology, Santa Cruz, CA, USA; 1 : 50), mouse anti-Nestin (Millipore, Billerica, MA, USA; 1 : 50), rabbit antiglial fibrillary acidic protein (GFAP, Dako, 1 : 200), rabbit anti-*β*III tubulin (Covance, 1 : 100), and goat anti-Myelin basic protein (MBP, Santa Cruz Biotechnology, 1 : 50) as primary antibodies. Cells were placed onto slides by means of cytospin and immunofluorescence assay was performed as previously described [21]. Fluorescent cell preparations were examined using a confocal microscope (Zeiss LSM 710). Noise reduction was achieved by Kalman filtering during acquisition. In order to perform a semiquantitative analysis, the number of immunoreactive cells was counted, evaluating at least 100 cells per sample over different areas of the slide.

### 2.6. RNA Extraction

RNA extraction from untreated and Pio 10 *μ*M treated cells for 96 hs was performed using the RNeasy Mini Kit (Qiagen), according to the manufacturer's protocol.

### 2.7. Real Time-PCR Array

RT^2^ Profiler PCR Arrays (Qiagen) were assessed on untreated and Pio 10 *μ*M treated cells for 96 hs in order to evaluate Pio effects on the expression of genes involved in Wnt pathway. RNA samples from treated and untreated cells were converted into first-strand cDNA using the RT^2^ First-Strand Kit (Qiagen). Then, RT^2^ Profiler PCR Arrays were assessed according to the manufacturer's protocol using custom array PCR, containing primers for 7 Wnt pathway-focused genes (*WNT1, FZD4, CTNNB1, EP300, CREBBP, TCF7,* and* MYC*) and for 2 housekeeping genes (*HPRT1, TBP*). Briefly the cDNA was mixed with an appropriate RT SYBR Green Mastermix. This mixture was aliquoted into the wells of the PCR Array and PCR was performed by means of a real time cycler (Applied Biosystems) properly programmed (1 cycle: 10 min 95°C, 40 cycles: 15 s 95°C, 1 min 60°C). Relative gene expression was determined using data from the real time cycler and the ΔΔCT method. The cut-off values for gene expression fold changes were established at +/− 1,5: values ≥ +1,5 indicated gene upregulation, while values ≤ −1,5 indicated gene downregulation. The gene expression fold changes data were obtained as mean values derived from two independent experiments.

### 2.8. Statistical Analysis

Statistical analysis was carried out performing *t*-test (MTT), Yates' Chi-square test (mitotic index), and Fisher's exact test (Immunofluorescence assay) on raw data, by means of Excel spreadsheet (Microsoft Office 2013, Microsoft Corporation). The critical level of significance was set at *p* < 0,05.

## 3. Results

### 3.1. GSC Lines Expressed PPAR*γ*

**Figure 1:**
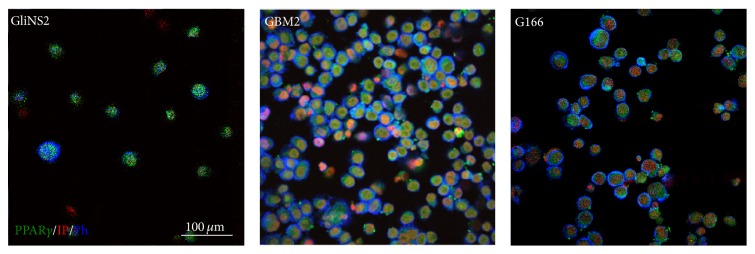
GSCs expressed PPAR*γ*. Selected images of immunofluorescence assays on GSCs. PPAR*γ* marker is in green; phalloidin is in blue and propidium iodide in red.

The expression of PPAR*γ* in the six GSC lines was evaluated by means of immunofluorescence assays. Results showed that all GSCs expressed PPAR*γ* at high levels (almost 100% of cells in all the GSC lines) (Figure 1).

### 3.2. Pio Exposure Variably Affected GSC Viability and Proliferation

**Figure 2:**
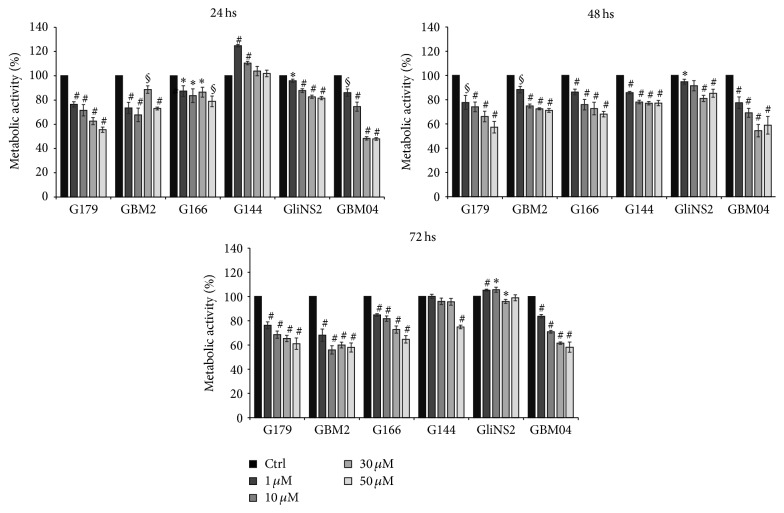
Pio effect on GSC viability. Cell viability was analyzed by MTT assay. Results represent the means from two different experiments performed at least in triplicate and are reported as percentage of drug-treated cells compared to matching untreated cells ± SEM. *t*-test on raw data: ^*∗*^ *p* < 0,05; ^§^ *p* < 0,01; ^#^ *p* < 0,001.

The effect of Pio on GSC viability was determined by means of the MTT assay (Figure 2).

After 24 hs of treatment GBM04 and G179 cell lines showed a drastic and dose-dependent reduction of metabolically active cells, compared to the matching untreated cells (*p* < 0,001). G166, GBM2, and GliNS2 cell lines highlighted a modest but significant decrease of the metabolic activity (*p* < 0,001). Contrariwise G144 cell line was resistant to Pio after this first time point showing even an increase in the percentage of metabolically active cells at all the drug concentrations tested.

Intriguingly, after 48 hs, increasing doses of Pio resulted in a progressive decrease of the metabolic activity in all the GSC lines, including G144.

After 72 hs Pio induced a dose-dependent decrease of the metabolic activity in G179, GBM2, and G166 cell lines (*p* < 0,001). In G144 cell lines the reduction was significant only after the exposure to Pio 50 *μ*M. An interesting behavior was highlighted in GliNS2 cell line which seemed to be less sensitive with the prolongation of the time of drug exposure, showing a slight decrease of the metabolic activity only after treatment with Pio 10 *μ*M (*p* < 0,05).

In all the GSC lines the reduction of the metabolically active cells was not time-dependent with the exception of GBM2.

**Figure 3:**
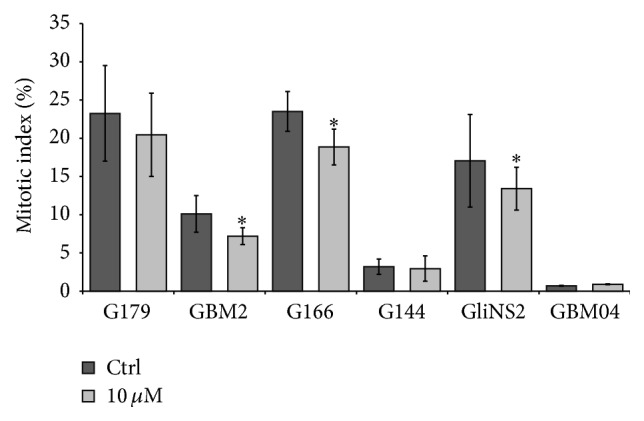
Pio effect on GSC proliferation. Cell proliferation was evaluated through the determination of the mitotic index. Results are reported as percentages resulting from means of two independent experiments ±SEM. Yates' Chi-square test on raw data: ^*∗*^ *p* < 0,05.

In order to study the effect of Pio on GSC proliferation, we evaluated the mitotic index (MI), a crucial parameter related to the tumor aggressiveness. We counted the number of metaphases per 1000 nuclei after exposure to Pio 10 *μ*M, the minimum concentration inducing a significant variation of the metabolic activity (Figure 3). Pio administration for 48 hs generally determined a significant decrease of MI in GBM2, G166, and GliNS2 cell lines, compared to matching untreated cells. G179 cell line showed a faint decrease of MI, but it was not statistically significant. In G144 and GBM04 cell lines the proliferation rate was extremely low even in the untreated cells. This feature prevented us from observing significant change in MI in these two cell lines.

### 3.3. Pio Did Not Induce Any Morphological Change in GSCs

**Figure 4:**
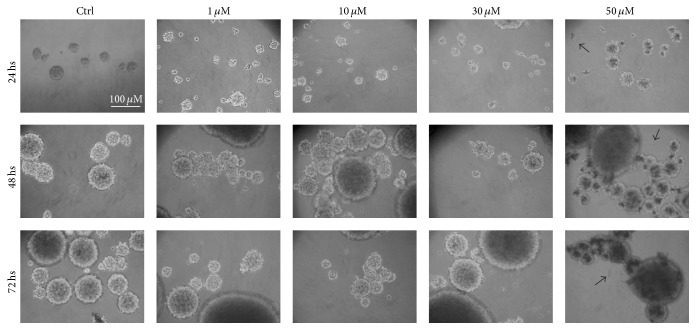
Representative images of GSC morphology after treatment with increasing concentrations of Pio for 24, 48, and 72 hs. Generally, Pio did not induce any relevant morphological change. Images of G144 cells show that GSCs still grow as neurospheres in nonadherent conditions after drug exposure. Black arrows indicate insoluble threadlike precipitates, which can also be observed on the cell surfaces.

GSC morphology analysis highlighted that both untreated and Pio-treated GSCs grew in nonadherent growth pattern as neurospheres, forming colonies varying in size. Generally Pio administration did not induce any relevant modification in cellular shape (Figure 4). However, at the highest concentration tested (50 *μ*M), Pio produced fusiform precipitates and showed a prevailing cytotoxic effect, especially after 48 hs and 72 hs.

### 3.4. Pio Effect on the Expression Levels of Stemness and Differentiation Markers

**Figure 5:**
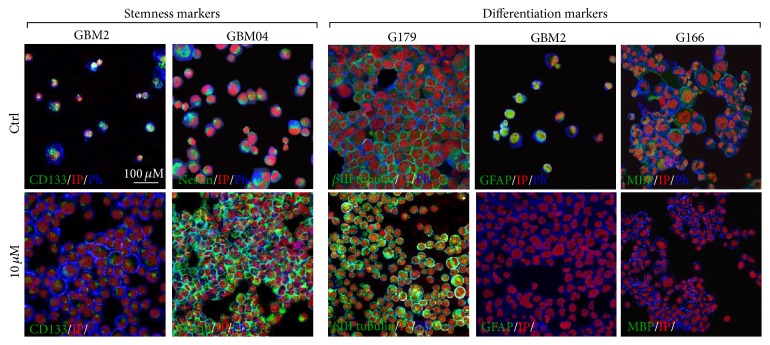
Selected representative images of immunofluorescence assays for stemness and differentiation markers. Immunofluorescence assays were performed on untreated (Ctrl) and 10 *μ*M Pio-treated GSCs for 72 hs. Each specific maker is in green; phalloidin is in blue and propidium iodide in red.

The prodifferentiating ability of Pio was evaluated through the expression analysis of selected stemness (CD133 and Nestin) and differentiation markers (GFAP, *β*III tubulin, and MBP) that had been previously used in the literature [3, 18, 22, 23], comparing cells untreated or treated with Pio 10 *μ*M, the minimal effective concentration, for 72 hs. Representative images are reported in Figure 5. The data overview showed that GSCs expressed both stemness and differentiation markers at variable levels (Table 1). Generally, Nestin was expressed at medium or high intensity in all untreated cell lines (range: 75,4–99,2%). CD133 immunoreactive cells were found in a large proportion of untreated GSCs in 5/6 cell lines (63–100%). Only G166 cell line showed a low expression of this marker (only 7,3% of CD133+ cells).

Pio treatment caused a reduction in the percentage of cells positive for at least one stemness marker in 3/6 lines. G166 showed a decrease in CD133+ cells (from 7,4% to 0%). GBM2 and GliNS2 showed a relevant decrease in Nestin immunoreactive cells from 75,4% to 0% and from 79,6% to 50%, respectively. GBM2 displayed also a slight but not statistically significant decrease in CD133 positive cells. Both these two lines after Pio administration showed also a remarkable reduction of cells positive for all the differentiation markers. Contrariwise G144 and GBM04 cell lines highlighted an increase in the percentage of cells positive for at least one stemness marker.

As regards the differentiation markers, G144 and G166 showed an increase in the percentage of cells immunoreactive for *β*III tubulin (resp., from 45,8% and from 25,9% to 100%) and GFAP (resp., from 82,5% and from 83,9% to 100%) and a reduction of cells positive for MBP (from 100% to 0%), while 100% of GBM04 cell were positive to all the differentiation markers after Pio treatment. In G179 Pio treatment caused a change only in the percentage of cells positive for MBP (from 0% to 100%).

### 3.5. Pio Mildly Modulated Wnt Signaling Pathway

The Wnt signaling expression profile was performed using custom PCR arrays on untreated and 10 *μ*M Pio-treated cells for 96 hs exploring the expression variations of 7 genes.

Results showed that Pio was able to mildly modulate Wnt signaling pathway in 3 out of 6 GSC lines (Table 2). In particular, G179 and G144 showed a downregulation of the ligand* WNT1*. G179 highlighted also an upregulation of the coactivators* EP300* and* CREBBP*. In GBM2 there was an evident increase in the expression levels of* WNT1, TCF7,* and the downstream oncogene* MYC*.

## 4. Discussion

PPAR*γ* is a ligand-activated nuclear transcription factor of the PPAR receptors superfamily indicated as a promising target for the development of new therapeutic approaches [4]. A number of studies have reported that the activation of PPAR*γ* by means of natural and synthetic ligands has chemotherapeutic effects [10, 24, 25].

In the present study we investigated the effect of Pioglitazone (Pio), a PPAR*γ* agonist commonly used in clinic for the treatment of type II diabetes mellitus, on six GSC lines isolated from GBM. Despite improvement in treatment outcome, GBM patients are still characterized by poor prognosis [1] and GSCs have been demonstrated to play a key role in tumor initiation and relapse following standard therapies [2, 3]. Accordingly, the identification of new therapeutic strategies GSC-targeted seems to be unavoidable for GBM eradication, and PPAR*γ* agonists could be a great bet [26, 27].

First of all we verified that PPAR*γ* was expressed in all the GSC lines, in agreement with what had been previously reported [28]. Afterwards we investigated if Pio administration would be able to affect cell viability and proliferation, as one of the hallmarks of cancer is the proliferative advantage over normal tissue.

MTT assay and the mitotic index evaluation highlighted a high variability in response to Pio exposure across our six GSC lines, identifying only a subgroup of Pio-sensitive GSC lines. This finding mirrors the well-known interpatient heterogeneity that characterizes this type of tumor. Large-scale genomic analyses have already revealed that GBM intertumoral molecular heterogeneity might account for differences in patient sensitivity to therapy and prognosis [29, 30]. Accordingly, also a previous study by our group showed a remarkable genomic and epigenomic variability in our GSCs [20]. As regards Pio treatment G179, GBM2, G166, and GBM04 showed the most evident reduction of cell viability and proliferation, which may be due to different mechanisms, such as the upregulation of proapoptotic protein or the arrest of cell cycle progression, as previously described [11–13, 31].

Afterwards, we evaluated if Pio treatment could cause a differentiation-like process in our GSCs inducing changes in cell morphology or in the expression of stemness and differentiation markers. A number of studies have suggested that PPAR*γ* agonists can modulate the differentiation of NSCs or GSCs downregulating the expression of stemness genes or increasing the expression of glial cell markers [32–34]. Contrariwise to what have been previously shown, we did not observe neither an alteration in cell morphology nor an evident differentiation signature in our GSCs. In fact after Pio exposure GSCs continued to grow as neurospheres and retained the coexpression of both stemness and differentiation markers, observed also in the untreated cells. However, this marker coexpression in cultured neurospheres is not surprising as it reflects the intratumor heterogeneity observed in patients: each tumor can present a complex hierarchical organization of distinct subclones with diverse morphologies and biological behaviors [30]. Moreover, a recent study has also demonstrated that in an individual tumor multiple subpopulations of GSCs can coexist characterized by distinct surface marker expression and single-cell molecular profiles relating to distinct bulk tissue molecular subtypes [35].

At last, we evaluated Pio effect on the expression of Wnt related genes because a key feature of GSCs is the aberrant activation of embryonic signaling pathways [15, 36] and previous studies had demonstrated that Pio was able to downregulate *β* catenin in glioma cells [17]. The analysis of the expression levels of 7 genes (*WNT1, FZD4, CTNNB1, EP300, CREBBP, TCF7,* and* MYC*) showed that Pio slightly modulated Wnt pathway in our GSCs and interestingly it did not alter *β* catenin levels. This finding is in net contrast with previous studies, which reported that Pio was able to decrease expression level of *β* catenin protein [17, 37] or downregulate Wnt/*β* catenin signaling pathway downstream transcriptional target genes [38].

## 5. Conclusions

In conclusion, this work demonstrated that the potential of the synthetic PPAR*γ* agonist Pio as a novel promising therapy for glioma is not so obvious. Pio effect was highly heterogeneous among different GSC lines. It reduced cell viability and proliferation only in a subset of GSC lines, suggesting that further studies about the mechanisms involved in the antineoplastic effects of PPAR*γ* agonists are needed. Interestingly, Pio did not affect cell morphology nor induced any differentiation-like process and Wnt/*β*-catenin signaling pathway seemed not to be the main mediator for Pio to suppress GSCs growth.

## Figures and Tables

**Table 1:** Expression of stemness and differentiation markers in GSC lines in response to Pio treatment.

GSC lines	Treatment	CD133	Nestin	*β*III tubulin	GFAP	MBP
		%	%	%	%	%
GBM2	Untreated	62,96	75,44	19,27	63,81	100
	Pio 10 *μ*M 72 hs	50	0^*∗∗∗*^	0^*∗∗∗*^	0^*∗∗∗*^	0^*∗∗∗*^
GBM04	Untreated	86,29	96,58	76,78	86,08	0
	Pio 10 *μ*M 72 hs	100^*∗∗∗*^	100	100^*∗∗∗*^	100^*∗∗∗*^	100^*∗∗∗*^
G144	Untreated	78,38	91,35	45,79	82,46	100
	Pio 10 *μ*M 72 hs	100^*∗∗∗*^	100^*∗*^	100^*∗∗∗*^	100^*∗∗∗*^	0^*∗∗∗*^
G166	Untreated	7,34	78,3	25,92	83,9	100
	Pio 10 *μ*M 72 hs	0^*∗*^	100^*∗∗∗*^	100^*∗∗∗*^	100^*∗∗∗*^	0^*∗∗∗*^
G179	Untreated	100	99,17	100	98,21	0
	Pio 10 *μ*M 72 hs	100	100	100	100	100^*∗∗∗*^
GliNS2	Untreated	100	79,63	71,43	93,64	100
	Pio 10 *μ*M 72 hs	100	0^*∗∗∗*^	0^*∗∗∗*^	16,2^*∗∗∗*^	0^*∗∗∗*^

Fisher's exact test on raw data: ^*∗*^ *p* < 0,05; ^*∗∗∗*^ *p* < 0,001.

**Table 2:** Fold regulation and trend of the expression variations of 7 Wnt pathway-focused genes in response to Pio treatment.

	G179		GBM2		G166		G144		GliNS2		GBM04	
	F.R.	Trend	F.R.	Trend	F.R.	Trend	F.R.	Trend	F.R.	Trend	F.R.	Trend
WNT1	−3,490	↓	8,273	↑	1,015	=	−2,311	↓	1,169	=	−1,266	=
FZD4	1,159	=	1,213	=	−1,120	=	1,087	=	−1,017	=	1,151	=
CTNNB1	−1,071	=	1,025	=	−1,288	=	1,471	=	−1,117	=	1,022	=
TCF7	−1,249	=	1,683	↑	−1,147	=	−1,369	=	1,047	=	−1,003	=
MYC	−1,052	=	3,216	↑	1,452	=	−1,058	=	1,065	=	−1,040	=
EP300	1,561	↑	−1,228	=	1,097	=	1,091	=	−1,058	=	−1,077	=
CREBBP	1,539	↑	−1,497	=	1,172	=	1,219	=	−1,050	=	1,101	=

Gene expression levels in GSCs were evaluated by means of RT-PCR after Pio 10 *μ*M exposure for 96 hs. F.R.: fold regulation; ↑: upregulated; ↓: downregulated; =: unchanged.
