# Correction to “Pioglitazone Effect on Glioma Stem Cell Lines: Really a Promising Drug Therapy for Glioblastoma?”

**DOI:** 10.1155/ppar/9796467

**Published:** 2026-07-01

C. Cilibrasi, V. Butta, G. Riva, and A. Bentivegna, “Pioglitazone Effect on Glioma Stem Cell Lines: Really a Promising Drug Therapy for Glioblastoma?,” *PPAR Research*, vol. 2016 (2016). https://doi.org/10.1155/2016/7175067.

In the article titled “Pioglitazone Effect on Glioma Stem Cell Lines: Really a Promising Drug Therapy for Glioblastoma?,” there was an error in Figure 5. Specifically, an incorrect image was selected for the cells in the control group stained for CD133/IP/Ph as it represents untreated GliNS2 cells stained for CD133 as reported in a previous publication by the same author group [1]. The error was introduced by the authors during figure assembly and Figure 5 should be corrected as follows:

**Figure 5:**
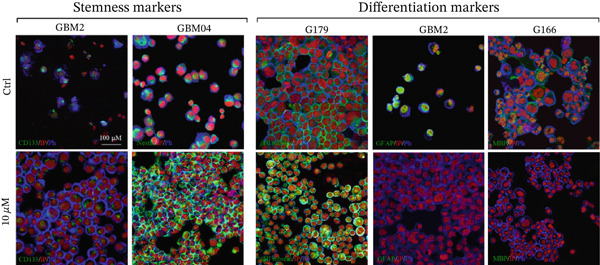
Selected representative images of immunofluorescence assays for stemness and differentiation markers. Immunofluorescence assays were performed on untreated (ctrl) and 10 *µ*M Pio‐treated GSCs for 72 h. Each specific maker is in green; phalloidin is in blue and propidium iodide in red.

We apologize for these errors.
